# Oncological Outcome of Node-Positive Oral Squamous Cell Carcinomas Treated With Selective and Comprehensive Neck Dissection

**DOI:** 10.1155/2024/9543897

**Published:** 2024-07-11

**Authors:** Jan Oliver Voss, Lea Freund, Felix Neumann, Kerstin Rubarth, Kilian Kreutzer, Steffen Sander, Evelyn Golembiewski, Friedrich Mrosk, Christian Doll, Carsten Rendenbach, Max Heiland, Steffen Koerdt

**Affiliations:** ^1^ Department of Oral and Maxillofacial Surgery Charité-Universitätsmedizin Berlin Corporate Member of Freie Universität Berlin Humboldt-Universität zu Berlin and Berlin Institute of Health, Augustenburger Platz 1 13353, Berlin, Germany; ^2^ Berlin Institute of Health (BIH), Anna-Louisa-Karsch-Straße 2 10178, Berlin, Germany; ^3^ Charité-Universitätsmedizin Berlin Corporate Member of Freie Universität Berlin and Humboldt-Universität zu Berlin Institute of Biometry and Clinical Epidemiology Charitéplatz 1 10117, Berlin, Germany; ^4^ Charité-Universitätsmedizin Berlin Corporate Member of Freie Universität Berlin and Humboldt-Universität zu Berlin Institute of Medical Informatics, Charitéplatz 1 10117, Berlin, Germany; ^5^ Charité Comprehensive Cancer Center Charité-Universitätsmedizin Berlin Corporate Member of Freie Universität Berlin Humboldt-Universität zu Berlin and Berlin Institute of Health, Virchowweg 23 10117, Berlin, Germany; ^6^ Department of Radiation Oncology Charité-Universitätsmedizin Berlin Corporate Member of Freie Universität Berlin Humboldt-Universität zu Berlin and Berlin Institute of Health, Augustenburger Platz 1 13353, Berlin, Germany

**Keywords:** comprehensive neck dissection, head and neck cancer, lymph node metastases, neck dissection, oral squamous cell carcinoma, selective neck dissection

## Abstract

Selective neck dissection (SND) is the treatment of choice in patients with oral squamous cell carcinomas (OSCCs) and clinically node-negative necks (cN0). The treatment of patients with positive-staged necks (cN+) includes SND as well as comprehensive neck dissection (CND). The clear benefit of one or the other remains under debate. We aim to address this lack of clarity by analysing patients with OSCC staged with clinically node-positive necks, treated with either CND or SND using a level-by-level approach. This retrospective study included patients diagnosed with OSCC with clinically (cN+) and pathologically (pN+) positive cervical lymph nodes (LNs) with clear neck level categorization during the years 2010–2019. In total, 74 patients were analysed. Cox regression analysis found no significance for the type of ND being an independent risk factor, neither for overall survival (OS) nor for disease-free survival (DFS). Regional recurrence of CND cases (5.77%) was comparable to SND cases (9.09%). For OS, extracapsular spread (ECS) and male sex were identified as independent risk factors with poorer outcome. pT-stage and ECS were found to be independent risk factors for DFS. The results of this study suggest that both CND and SND may be viable treatment options for certain patients with OSCC pN+.

## 1. Introduction

Oral squamous cell carcinoma (OSCC) has a high potential to spread into locoregional lymph nodes (LNs), especially in patients with locally advanced tumors [1]. Up to 34% of patients with OSCC initially categorized with a clinically node-negative neck (cN0) may have occult metastases detected during histological workup, mainly in Robbins levels I–III [2]. In locally advanced stages, the risk of overall (cervical) LN metastasis (LNM), particularly in the more latero-caudally located levels of the neck (Robbins levels IV and V), is higher and associated with worse overall survival (OS) of the patient [3, 4]. Neck dissection (ND), which can be carried out to different extents depending on the preoperative staging, is an elementary part of the surgical treatment of OSCCs, including selective ND (SND), comprehensive ND (CND), and even radical ND (RND).

For patients with OSCC and no clinically apparent nodal disease in preoperative staging (cN0), SND is the treatment of choice and includes levels I–III as described by Robbins [5–7]. Algorithms for neck treatment in cN0 necks have been established and represent an important aspect of surgical oncologic treatment of OSCC [8].

CND is defined as the clearance of levels I–V with preservation of the internal jugular vein, and/or the spinal accessory nerve, and/or sternocleidomastoid muscle [9]. CND has been established as a standard procedure in radiological and/or clinically positive-staged (cN+) necks/advanced nodal disease in a stagewise adapted clinical concept.

Although the SND treatment regime in cN0 necks is accepted worldwide, the extent of ND, especially in cN+ necks, remains under debate [10]. However, the extent of ND, as well as any adjuvant/nonsurgical treatment regimen, can influence health-related quality of life [11–14]. Shoulder dysfunction including scapula flaring, shoulder drop, and limited arm abduction attributed to spinal accessory nerve impairment, phrenic nerve injury, and chylous leakage, has been associated with the extent of ND [15–18]. Therefore, a less extensive surgical treatment regime while maintaining oncologic control would be preferable.

This retrospective study is aimed at evaluating the efficacy and oncological outcome of patients initially staged as cN+ with cervical LNMs in pathological workup (pN+) treated with SND compared to CND, with a particular focus on the level of LN involvement.

## 2. Patients and Methods

### 2.1. Ethical Agreement

The study was conducted in compliance with applicable guidelines and regulations. Since this was a retrospective study, informed consent was not required. Ethical approval for data collection and publication was granted by the institutional review board of the Charité–Universitätsmedizin Berlin (EA1/077/20).

### 2.2. Study Design

This retrospective analysis focused on patients diagnosed with primary squamous cell carcinoma of the oral cavity who presented with cervical LNMs and were treated at the Department of Oral and Maxillofacial Surgery, Charité–Universitätsmedizin Berlin, from 2010 to 2019. Patients included in the study had undergone level-by-level NDs to enable precise mapping of LNMs to specific neck regions.

Eligibility was determined using International Classification of Diseases (ICDs)-10 codes, clinical and pathological TNM classifications, and staging from the Union for International Cancer Control (UICC, 8th Edition). Key factors analysed included LN status (total number removed, total number of cervical LNMs), localization (side of the neck, affected Robbins level I–V), extracapsular spread (ECS), primary surgical treatment, final therapy documentation (surgery, radiation, and with or without chemotherapy), adjuvant chemotherapy, treatment volume (anatomical region of primary tumor and/or LN levels), and total radiation doses. The study also considered tumor recurrence, regional and distant metastasis, time to death, tumor-related death, disease-free survival (DFS) (date of surgery to last contact, local recurrence, nodal relapse, distant metastasis, or tumor-related death), and OS. Patients lacking clear information about the involved neck level of LNMs and those initially staged as cN0 were excluded to prevent selection bias.

**Figure 1:**
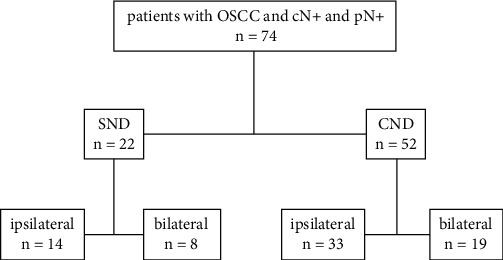
Schematic illustration of the study group. Abbreviations: pN+ = pathologically positive cervical lymph nodes; cN+ = clinical evidence of cervical nodal disease; SND = selective neck dissection (levels I–III); CND = comprehensive neck dissection.

The study cohort consisted of 74 patients with positive LNs identified during histopathological examination, all of whom were initially staged as cN+ (Figure 1). Patients received either SND (*n* = 22) or CND (*n* = 52), performed either unilaterally or bilaterally. For bilateral ND with unilateral metastasis, only the affected side was included. In cases of bilateral ND with bilateral metastasis, the mean value for both sides was used. Subgroup analyses compared SND and CND.

LN density (LND) was defined as the total number of metastatic LNs and was calculated separately for SND and CND. LN yield (LNY) described the total count of LNs removed on one side of the neck and was specified separately for SND and CND. LN ratio (LNR) was defined as the ratio of the number of metastatic LNs to the total number of LNs removed, calculated separately for SND and CND. Indications for postoperative radio(chemo)therapy included ECS, ≥ pN1, and close resection margins, as well as pT3/4.

### 2.3. Procedural Definitions

In SND, cervical LN dissection encompasses levels I–III (Ia, Ib, IIa, IIb, and III), preserving crucial nonlymphatic structures such as the internal jugular vein, spinal accessory nerves, and sternocleidomastoid muscle. Conversely, CND involves the dissection of cervical LNs across levels I–V (Ia, Ib, IIa, IIb, III, IV, Va, and Vb), following procedural guidelines outlined by Holmes in 2008 [9].

### 2.4. Statistical Analysis

Data were collected in Microsoft Excel (Microsoft Corporation, Redmond, WA, USA) and analysed with SPSS Statistics Version 27.0 (IBM Corporation, Armonk, NY, USA). The results were considered statistically significant at *p* < 0.05. Due to the exploratory characteristic of the study, no adjustment of *p* values for multiple testing was conducted. Hence, *p* values were not interpreted as confirmatory. Normally distributed continuous data were analysed using Student's *t*-test, with means and standard deviations (SDs) calculated for metric data, and medians and interquartile ranges (q1 and q3) for count data. Cox regression with stepwise conditional backward selection included variables such as age, sex, ECS status, pathological T-status, adjuvant therapy, and ND type. Hazard ratios (HRs) with 95% confidence intervals (CIs) were calculated. ROC curves defined cut-offs using Youden's index. Kaplan–Meier analyses for OS and DFS were performed, with log-rank tests to examine relationships between categorical variables and survival outcomes. OS was defined as the time from surgery to death or last follow-up, and DFS as the time from surgery to tumor recurrence, metastasis, death, or last follow-up.

## 3. Results

### 3.1. Patients and Clinical Data

A total of 74 patients, 29 females (39.2%) and 45 males (60.8%), were included in this retrospective study. The mean ± SD age at diagnosis was 63.68 ± 10.7 years (range: 40–89 years). Median follow-up was 22.60 months (0.16–114.79, *q*1 = 7.65, *q*3 = 46.93). Clinical and histopathological TNM and UICC status are summarized in Table 1.

In total, 29.7% of all study patients (*n* = 22) received SND either ipsilaterally (*n* = 14) only or bilaterally if the tumor was approaching the midline (*n* = 8), and 70.3% underwent CND (*n* = 52), of which 33 patients received CND ipsilaterally and 19 bilaterally.

### 3.2. Pattern of LNMs

A median of 30.0 (range: 2–78, *q*1 = 20.75, *q*3 = 45.0) LNs were removed during surgery, and a median of 2.0 (range: 1–12, *q*1 = 1.0, *q*3 = 3.5) LNMs were detected by postoperative histopathological analysis. These values were grouped according to the type of ND (Table 2) and showed a significantly higher number of metastases in patients treated with CND.

The evaluation of the distribution of LNMs was carried out according to the neck side. In cases of bilateral ND with LNMs on both sides and the same extent of LN removal (SND or CND), the mean value for both sides was used. The mean total number of LNs resected for SND was 30.73 and for CND was 35.03. LNMs were located ipsilaterally in 83.8% (*n* = 62) of all patients, whereas LNMs could be found on both sides of the neck in another 16.2% (*n* = 12) of all patients. Patients with contralateral LNMs showed nodal spread mainly in levels I–III. Contralateral LNM at level IV was only present in one patient, whereas none were found at level V. ECS was detected in 44.6% (*n* = 33) of all patients.

**Figure 2:**
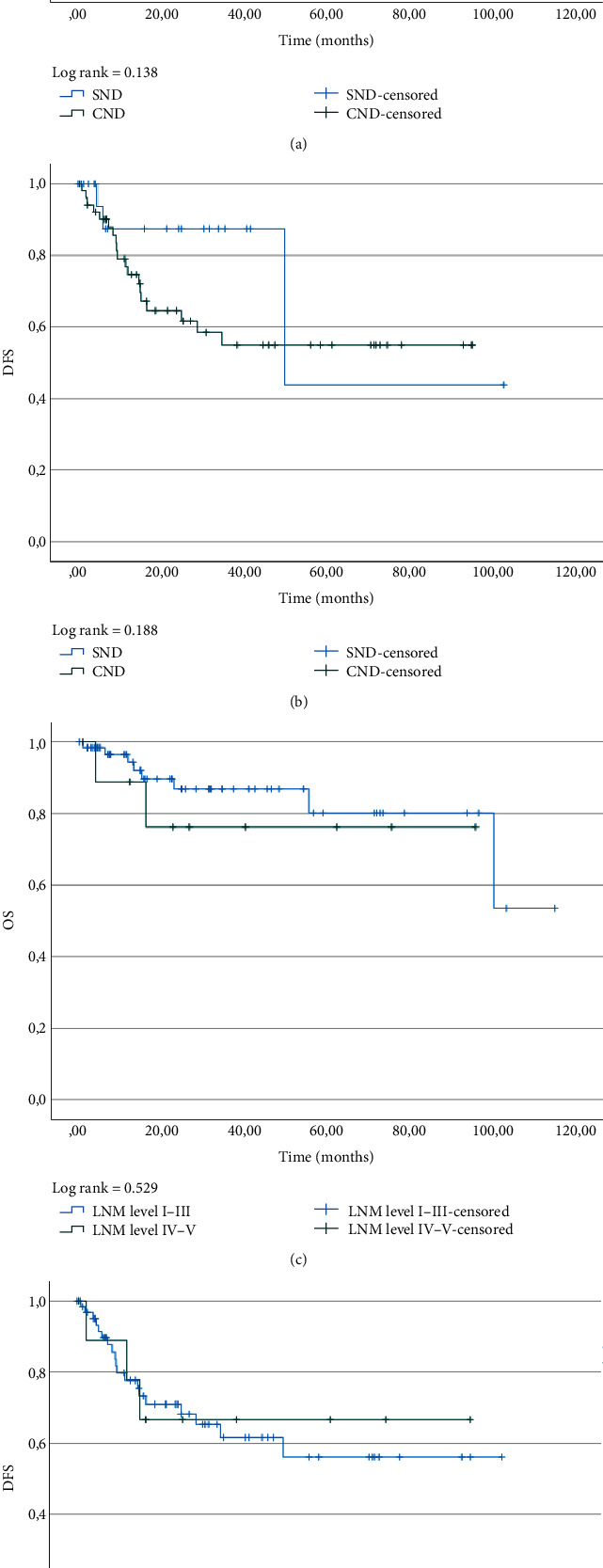
Kaplan–Meier curves for (a) OS and (b) DFS in groups with selective neck dissection (SND) levels I–III (either uni- or bilaterally; *n* = 22) and for those who underwent comprehensive neck dissection (CND) (*n* = 52). Kaplan–Meier curves for (c) overall survival (OS) and (d) disease-free survival (DFS) in groups with lymph node metastases (LNM) at levels I–III only (*n* = 64), and those with LNMs at levels I–III and levels IV and V (*n* = 10).

In a comparison of patients who received SND and those who underwent CND, there were no significant differences for OS and DFS (Figures 2(a) and 2(b)). Also, no significant differences could be detected in terms of OS and DFS in groups with LNMs at levels I–III and those with metastases at levels I–III and levels IV and V (Figures 2(c) and 2(d)). The distribution of patients with LNMs (ipsilaterally or bilaterally in cases of cancers in the anterior floor of the mouth, or extending across the midline) according to neck levels (patients with LNMs at multiple levels were counted several times) is shown in Table 3.

The median LNY was 28.5 (*q*1 = 20.75; *q*3 = 40.63) for SND and 32.0 (*q*1 = 19.5; *q*3 = 46.75) for CND. The median LNR was 0.05 (*q*1 = 0.03; *q*3 = 0.10) for SND and 0.09 (*q*1 = 0.04; *q*3 = 0.13) for CND. The optimal cut-off points for LNY and LNR are displayed in Table 4. Most cut-off values showed low areas under the curve (AUC). The cut-off points for LNY in patients with SND showed a larger AUC for OS and DFS, and also the cut-off points for LNR in patients with SND and CND for DFS. Overall, after performing survival analysis, none of the cut-off points showed statistically significant differences.

**Figure 3:**
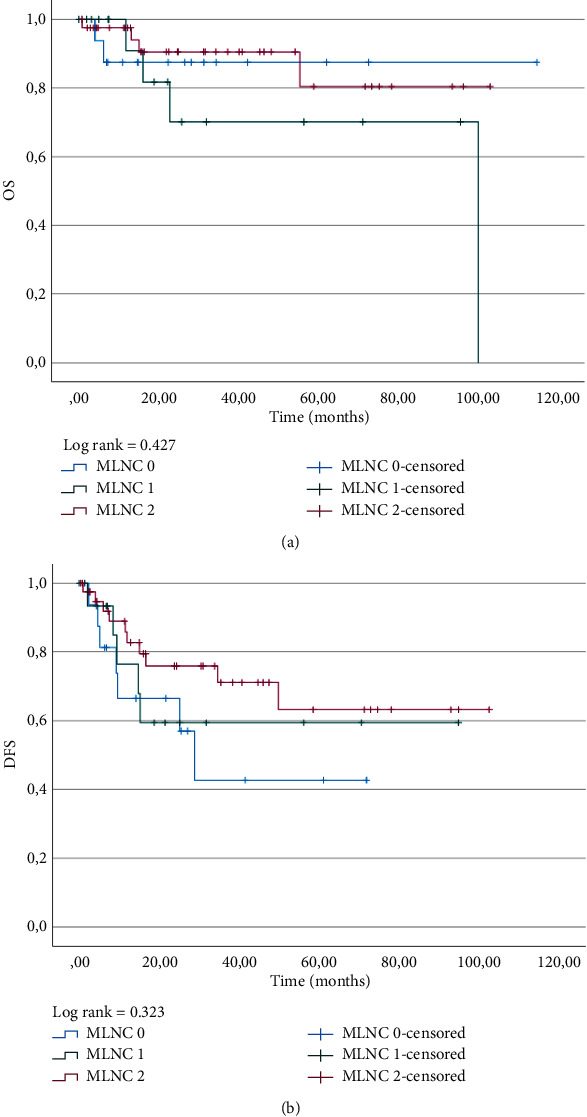
Kaplan–Meier curves for (a) OS and (b) DFS regarding the metastatic lymph node clearance (MLNC) score.

Metastatic LN clearance (MLNC) was calculated using the LNY and LNR cut-off values (minimum score = 0, maximum score = 2) [19]. Survival analysis was performed for OS and DFS, using the MLNC score to group patients (Figure 3). The Kaplan–Meier curves showed a tendency for better long-term DFS for patients with a higher MLNC score, whereas for OS, the curve for low MLNC showed a slight tendency for better survival. Nevertheless, there was no statistical significance.

### 3.3. Highest Positive Anatomical Level (HPAL)

The HPAL was determined for all included cases. The results are displayed in Table 5.

Patients with LNMs at levels I and II were compared to patients with LNMs at level III, according to the occurrence of LNMs at higher levels (IV or V). If patients presented with LNMs at multiple levels, the highest was used. The results are shown in Table 6. Patients with LNMs at level I or II had LNMs at level IV (*n* = 3) or V (*n* = 2) in 11.90% of cases, whereas patients with LNMs at level III had LNMs at level IV (*n* = 1) or level V (*n* = 4) in 15.63% of cases. Thus, the risk for LNMs at higher levels was higher in patients with LNMs at level III compared to patients with LNMs at level I and/or II.

### 3.4. pN Status

The histological LN status was evaluated for SND and CND cases. The results are shown in Table 7.

**Figure 4:**
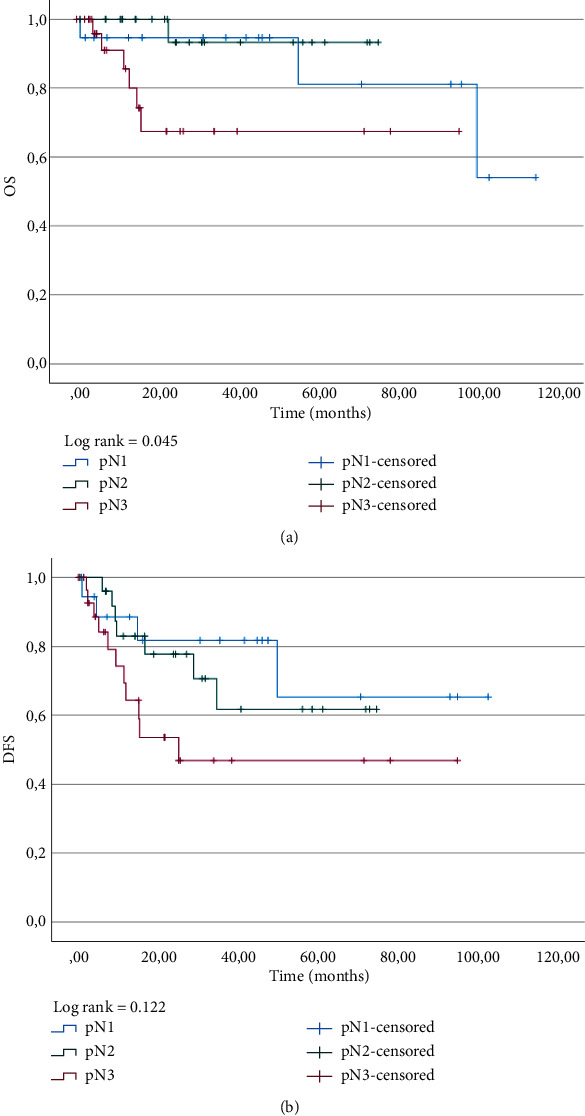
Kaplan–Meier curves for (a) OS and (b) DFS according to histological lymph node status (pN).

Survival analysis was performed for OS and DFS according to pN status (Figures 4(a) and 4(b)). There was a significant difference between groups for OS (*p* = 0.045) whereas there was none for DFS (*p* = 0.122).

### 3.5. Regional Control and Risk Factors

In terms of regional recurrence, 5.77% (3/52) of all patients who received CND showed LNMs during follow-up examinations. This rate was slightly lower than the recurrence rate after SND, which was 9.09% (2/22). Regarding overall recurrence, three (13.64%) of the patients who received SND presented with recurrences, which is a significantly lower rate compared to patients with recurrence who received CND (*n* = 16; 30.77%). Regional and overall recurrence are displayed in Table 8.

Cox regression analysis using the conditional stepwise backward selection method was performed to identify risk factors for poorer OS and DFS. Sex, age, pT-stage, ECS, adjuvant therapy, and the type of ND were included and tested. For OS, ECS and male sex were identified as an independent risk factor for poorer outcome. For DFS, the identified risk factors were advanced pT-stage and ECS. The type of ND was not identified as a significant independent risk factor for OS or for DFS. Cox regression analyses are summarized in Table 9.

### 3.6. Adjuvant Therapy

Based on the pathological results and patient conditions, the postoperative interdisciplinary oncological conference recommended adjuvant therapy in 74.3% of all patients (*n* = 55; 40 CND and 15 SND). In 25.7% of the cases (*n* = 19; 12 CND and 7 SND), no adjuvant therapy was recommended either because of impaired general health or because patients declined adjuvant therapy. Adjuvant radiation therapy was performed with a mean total dose of 60.90 ± 9.25 gray (Gy) to the tumor bed and/or ECS region and 52.13 ± 7.40 Gy to the elective cervical LN levels. Bilateral radiation therapy of cervical LNs was administered in 48 cases (90.57%), and in 5 cases (9.43%), unilateral radiation therapy was performed. In total, 27 patients received concurrent chemoradiation with cisplatin (*n* = 23, 31.1%), carboplatin (*n* = 2, 2.7%), mitomycin (*n* = 1, 1.4%), or erbitux (*n* = 1, 1.4%).

## 4. Discussion

SND is a widely accepted treatment regime in early-stage disease with cN0 neck [5, 6, 20]. However, its extension to levels further caudally with the preservation of important nonlymphatic structures, in terms of a CND in cases with preoperative evidence of LNMs, remains controversial, with unpredictable outcomes [21]. A nationwide survey of the German Association of Oral and Maxillofacial Surgery reported that 69.74% of participating hospitals included neck levels IV and V in patients with node-positive necks [22].

From a technical point of view, the extension from SND to CND is associated with increased postoperative morbidity, including wound healing disorders, a higher rate of damage to important nervous structures, such as the spinal accessory nerve, lymphatic fistulas due to damage to the thoracic duct, and other rare complications [11, 23]. According to the guidelines, postoperative adjuvant radiation or chemoradiation is the preferred treatment for cases of pathological LNMs [24]. SND was considered a valid option in selected patient cases with nodal disease in a literature review analysing the feasibility of SND of levels I–III in patients with preoperative staged cN+ necks [25]. In a meta-analysis, Liang et al. concluded that the clinical outcome in cN+ OSCC patients treated with SND of levels I–III in combination with adjuvant therapy was comparable to those undergoing comprehensive ND [26].

A retrospective analysis of patients with an OSCC and cN+ neck treated with either CND or RND reported an occult metastasis rate below 5% at level V. However, a statistically significant association was noted between level V metastases and a positive N stage above N2b. The authors concluded that the level V LN pads might be preserved in these patients with a clinically N+ neck below the nodal stage N2a [27]. Similar results were reported by Shin et al. Here, a nodal staging above N2b was significantly related to a higher rate of level IV or V LNM in patients with OSCC and cN+. However, the authors stated that SND combined with adjuvant therapy achieved regional control and survival rates comparable to comprehensive ND in patients under the N stage of cN2a OSCC [28].

Similar results were reported in a retrospective analysis of cN+ and pN+ OSCC patients comparing different types of ND. In that analysis, Andersen et al. concluded that it would be reasonable to extend the indications for SND to patients with a higher stage of nodal disease (N2) with nonfixed nodes [29]. In a retrospective cohort study of pN+ OSCC patients by Zeng et al., no significant difference was reported between an SND group and the CND group in terms of a 3-year neck control rate or disease-specific survival rate [30].

In a different study, patients with oral cavity squamous cell carcinoma were prospectively evaluated for the prevalence of histopathological metastasis to level V nodes in cN0, cN1, and cN2 patients. The authors concluded that, with the exception of cN0 patients, SND including levels I–III would be a reasonable treatment option in patients with cN1 oral cavity cancer with level lb as the only site [12].

The prevalence of LNM at level V is generally higher in hypo- and oropharynx compared to oral cancers. Davidson et al. reported a prevalence of LNM at level V of 1% in patients with oral cancers [31].

Feng et al. analysed 637 patients with OSCC who were treated by different ND regimes and did not find skip metastases alone at level IV or V or any neck recurrence at level IV or V [32]. In this context, in a systematic review assessing the prevalence of level IV and/or V involvement or skip metastases in patients with clinically negative or positive necks and OSCC, the authors concluded that selective ND including levels I–III would be reasonable in selected patients with cN+ necks [33]. In our study, we observed that the majority of LNMs were situated at levels I–III and that there were no significant differences in OS or DFS with regard to the localization of LNM. The prophylactic value of CND was limited in our overall analysis, and neither the type of ND performed nor the initial staging of potential LNMs seemed to play a significant role in long-term follow-up. However, surgical decisions to perform CND instead of SND within our study were biased by cases with clinically obvious LNM.

We observed a crossing of survival curves (Figure 2) indicating a violation of the proportional hazard assumption, and concerns have been raised previously regarding whether the log-rank test is the correct type of analysis in this situation [34]. Therefore, due to the small number of included patients and thus the number of events, *p* values should be interpreted with caution.

From a surgical standpoint, the extension of a SND to a CND only seems to be inevitable with LNMs at levels IV/V, whereas extension in cN+ staged necks does not seem to have an impact on survival rates. Although the data in this current study is somewhat heterogeneous, a simple extension to a CND in cN+ cases does not account for the complexity of this question, keeping in mind that all ipsilateral levels should be included in the adjuvant radiation field.

Moreover, we recommend an adapted approach to neck management in cN+ cases. Following N-staging according to the TNM system, the localization of the LNMs should be considered. In addition to the number of LNMs, ECS adjacent to anatomical structures, and allocation to the ipsi- or contralateral neck site, the neck level also seems to be important in decision-making with regard to the extension of ND. A stagewise concept following initial staging might assist with the individual adaptation of the type of ND for oncologic use. Clinically apparent LNM at levels I–III appears to be sufficiently addressed by SND, whereas LNM at levels IV and V calls for an extension to CND. Considering the overall low number of LNMs at levels IV and V and the importance of adjuvant radiation with or without concomitant chemotherapy, prophylactic clearance of levels IV and V might not be justified. However, a prospective randomized controlled trial is necessary to prevent biased decisions based on retrospective analysis.

## 5. Conclusions

ECS and male sex were identified as independent risk factors for poorer overall survival, while pT-stage and ECS were found to be independent risk factors for DFS. The type of ND was not identified as a significant independent risk factor for overall survival or DFS. The results of this study suggest that both CND and SND may be viable treatment options for certain patients with OSCC pN+. Further research is needed to identify other risk factors that may impact outcomes in these patients.

## Figures and Tables

**Table 1:** Clinical and histopathological TNM and UICC status.

**Parameter**	**n** **(%)**
cT stage	
cTis	1 (1.4)
cT1	9 (12.2)
cT2	25 (33.8)
cT3	8 (10.8)
cT4a	20 (27.0)
cTx	11 (14.9)
cN stage	
cN1	32 (43.2)
cN2a	16 (21.6)
cN2b	13 (17.6)
cN2c	13 (17.6)
cN3	0 (0)
cM stage	
cM0	72 (97.3)
cM1	2 (2.7)
cUICC	
III	20 (27.0)
IVa	39 (52.7)
IVb	2 (2.7)
IVc	2 (2.7)
n/a	11 (14.9)
pT-stage	
pT1	12 (16.2)
pT2	24 (32.4)
pT3	15 (20.3)
pT4a	22 (29.7)
pT4b	1 (1.4)
pN stage	
pN1	19 (25.7)
pN2a	4 (5.4)
pN2b	17 (23.0)
pN2c	5 (6.8)
pN3a	0 (0)
pN3b	29 (39.2)
pM stage	
pM0	73 (98.6)
pMx	1 (1.4)
pUICC	
III	13 (17.6)
IVa	30 (40.5)
IVb	30 (40.5)
n/a	1 (1.4)

Abbreviation: n/a = not applicable.

**Table 2:** Evaluation of dissected lymph nodes according to type of ND.

	**n**	**Mean**	**SD**	**p** **value**
*LN total*				
SND	22	30.73	14.36	0.340^∗^
Unilateral	14	27.93	15.64	
Bilateral	8	35.63	11.01	
CND	52	35.03	17.54	
Unilateral	33	34.67	15.89	
Bilateral	19	35.66	20.55	
*LN metastases*				
SND	22	1.86	1.26	0.03^∗^
Unilateral	14	1.93	1.49	
Bilateral	8	1.75	0.76	
CND	52	2.91	2.04	
Unilateral	33	3.18	2.40	
Bilateral	19	2.45	1.08	

Abbreviations: CND = comprehensive neck dissection, LN = lymph node, ND = neck dissection, SD = standard deviation, SND = supraomohyoid neck dissection.

^*^Statistically significant at *p* < 0.05.

**Table 3:** Distribution of patients with lymph node metastases (ipsilaterally or bilaterally in cases of cancers in the anterior floor of the mouth, or extending across the midline) according to neck levels (patients with LNMs at multiple levels were counted several times).

**Level**	**IA**	**IB**	**IIA**	**IIB**	**III**	**IV**	**V**
*n*	2	51	36	9	34	5	6
%	1.40	35.66	25.17	6.29	23.77	3.50	4.20

**Table 4:** Cut-off values for LNY and LNR regarding OS and DFS, divided by mode of neck dissection.

**Parameter**	**Survival**	**Cut-off value**	**Sensitivity**	**Specificity**	**AUC**	**Log rank**
LNY (SND)	OS	18	0.91	1.0	0.91	0.48
LNY (SND)	DFS	18	0.95	0.67	0.77	0.10
LNY (CND)	OS	46	0.44	0.70	0.51	0.51
LNY (CND)	DFS	22	0.90	0.27	0.55	0.35
LNR (SND)	OS	0.06	1.0	0.57	0.57	0.48
LNR (SND)	DFS	0.05	1.0	0.53	0.60	0.10
LNR (CND)	OS	0.10	0.56	0.61	0.57	0.43
LNR (CND)	DFS	0.09	0.68	0.64	0.63	0.08

**Table 5:** Highest positive anatomical level showing lymph node metastasis.

**Highest positive anatomical level**	**I**	**II**	**III**	**IV**	**V**
*n*	15	22	27	4	6
%	20.3	29.7	36.5	5.4	8.1

**Table 6:** Distribution of LNMs at levels IV and V, depending on LNM pattern at levels I–III.

**Highest positive anatomical levels I–III**	**I–II**	**III**
*n*	42	32
*n* LNM level IV (%)	3 (7.14)	1 (3.13)
*n* LNM level V (%)	2 (4.76)	4 (12.50)

**Table 7:** Histological lymph node status according to type of neck dissection.

	**n**	**%**
SND	22	
pN1	10	45.45
pN2a	1	4.55
pN2b	3	13.64
pN3b	8	36.36
CND	52	
pN1	9	17.31
pN2a	3	5.77
pN2b	14	26.92
pN2c	5	9.62
pN3b	21	40.38

**Table 8:** Local recurrence and overall recurrence following SND versus CND.

	***n***	**%**
SND		
Overall	22	
Local recurrence	2	9.09
Overall recurrence	3	13.64
CND		
Overall	54	
Local recurrence	3	5.77
Overall recurrence	16	30.77

**Table 9:** Results of a Cox regression model for OS and DFS.

**Parameter**	**p** **value**	**HR**	**95% CI**
OS			
Sex	0.050	0.204	0.04–1.00
pT-stage	0.070	1.793	0.95–3.37
ECS	0.040	4.209	1.07–16.58
DFS			
pT-stage	0.016	1.631	1.10–2.43
ECS	0.035	2.540	1.07–6.04

## Data Availability

Access to data is restricted due to ethical concerns.
